# Heatstroke risk informing system using wearable perspiration ratemeter on users undergoing physical exercise

**DOI:** 10.1038/s41598-023-27492-9

**Published:** 2023-01-09

**Authors:** Hideya Momose, Mieko Takasaka, Tomomi Watanabe-Asaka, Moyuru Hayashi, Daisuke Maejima, Yoshiko Kawai, Toshio Ohhashi

**Affiliations:** 1 Department of Innovation of Medical and Health Sciences Research, Shinshu University School of Medicine, 3-1-1 Asahi, Matsumoto, 390-8621 Japan; 2 Division of Physiology, Tohoku Medical and Pharmaceutical University, Sendai, Japan

**Keywords:** Biotechnology, Physiology, Health care, Medical research

## Abstract

We constructed an informing system to users for the heatstroke risk using a wearable perspiration ratemeter and the users’ thirst responses. The sweating ratemeter was constructed with a capacitive humidity sensor in the ventilated capsule. The timing point for informing heatstroke risk was decided to change from positive to negative on the second derivative of sweating curve. In addition, a wearable self-identification and -information system of thirst response was constructed with a smartphone. To evaluate the validity of wearable apparatus, we aimed to conduct human experiments of 16 healthy subjects with the step up and down physical exercises. The blood and urine samples of the subjects were collected before and after the 30-min physical exercise. The concentrations of TP, Alb, and RBC increased slightly with the exercise. In contrast, the concentrations of vasopressin in all subjects remarkably increased with the exercise. In almost subjects, they identified their thirst response until several min after the informing for heatstroke risk. In conclusion, the wearable ratemeter and self-information system of thirst response were suitable for informing system of heatstroke risk. The validity of timing point for informing heatstroke risk was confirmed with changes in the thirst response and concentrations of vasopressin in blood.

## Introduction

Heatstroke has become a critical social concern in the world, especially in Japan owing to its wet and hot weather in summer. It is categorized as either passive or exertional depending on its causes^1^. The former is reported primarily among elderly persons whose ability to physiologically adjust to heat stress has become compromised^2^. In addition, children are also regarded as a population at risk. Children’s susceptibility to passive heatstroke is attributed to a high ratio of surface area to mass, an underdeveloped thermoregulatory system, small blood volume relative to body size, and a low sweating rate^3^.

In contrast, the latter is directly related to physical activity. Generally, athletes, firefighters, and agricultural workers are more concerned^4^. A substantial amount of sweating and wet skin are typical signs of exertional heatstroke, whereas, in passive heatstroke, the skin is usually dry, reflecting a characteristic decrease in the sweat-gland response and output in elderly people under heat stress. The skin may either be flushed, reflecting excessive peripheral vasodilation, or pale, indicating vascular collapse^1^. However, the pathophysiology of heatstroke and mechanism-based treatment approaches have not yet been elucidated. In particular, studies on new biomarkers that can significantly predict the outcomes of heatstroke have not yet been reported.

The sudomotor activities in human subjects involves the frontal operculum, hypothalamus, brainstem, spinal cord, sympathetic chain ganglia, peripheral nerve, and eccrine sweat glands^5–7^. Therefore, the patients in cardiovascular diseases with the troubles such as central or peripheral nervous system usually show the sign of hyperhidrosis or hypohidrosis^6^. Especially, the patient with severe myocardial infarction occurs palmar hyperhidrosis, resulting in cold hand^8,9^.

On the other hand, active palmar sweating in human subjects was confirmed to collaborate with neural activities of the limbic-cortical centers including the amygdala, hippocampus, and prefrontal cortex^8,9^. The sudomotor pathways from these centers run through the brain stem, spinal cord, and peripheral cholinergic sympathetic nerve fibers to the eccrine glands in palmar skin^8,9^. In fact, non-selective viral encephalitis in the amygdala in young patients did not induce active palmar sweating, although physiological palmar sweating was detected approximately 2 weeks after drug treatment^10^. We currently demonstrated that the faster spike in galvanic skin response (GSR) completely agreed with the starting point of active palmar sweating. Eyes closing and opening or self-awareness of drowsy significantly produced changes in baseline GSR and active palmar sweating, which may become useful tools for evaluating clearness or drowsiness in human subjects^11^.

Notably, a considerable amount of thermal sweating increases blood osmolality that stimulates thirst and vasopressin secretion through the activation of osmoreceptors in the hypothalamus or central osmoregulatory pathways^12,13^. However, the mechanism and pattern of thermal sweating and its contribution to thermoregulation in exertional heatstroke have not been elucidated.

Previously, we designed and constructed a new perspiration ratemeter using capacitance-typed humidity sensors and ventilated chambers circulated with airflow^14,15^. Compared with a previous ventilated capsule with a huge cylinder system to perfuse dry N_2_ gas, the original ratemeter shows a quick step-response and high sensitivity^14,15^.

To inform the risk to expose heatstroke to the user via a sound from the smartphone, we constructed (1) a new designed wearable perspiration ratemeter by modifying the original, (2) a wireless self-identification and self-information system with the smartphone for thirst response, and (3) a system that informs the heatstroke risk to the users. In addition, to evaluate the validity the timing point to inform automatically for the heatstroke risk to the user using a changing point of negative value on second differentiation of sweating curve, we conducted human experiments with a-30 min step up and down physical exercise and simultaneously recorded exercise-induced sweating on the neck and the thirst response. The changes in the concentrations of vasopressin, blood and urine samples, body weight, and heart rate before and after the exercise are measured to evaluate the relationship between the vasopressin release and the self-identification of thirst response related to the activation of osmoreceptors in the hypothalamus.

## Materials and methods

### Ethical approval

The study was approved by the Ethical Committee for Human Clinical Studies at the School of Medicine, Shinshu University (CRB3200010, approval no. 4445 on 6th August 2019). All study data and procedures were performed in accordance with the principles outlined in the Declaration of Helsinki. The study is registered in the WHO International Clinical Trial Registry Platform (13th/August/2022, https://www.who.int/clinical-trials-registry-platform: jRCT1032220270, Analysis of mental and thermal sweating in human subjects using galvanic skin response and domestic-made perspiration ratemeter).

### Construction of wearable perspiration ratemeter and self-information apparatus with a smartphone

Previously we constructed a high-sensitive perspiration ratemeter which is suitable for measuring active palmar sweating. Thus, a maximum value in the step response is obtained at approximately 0.63 s. The sensitivity of the electrical performance is 0.1/1 mg water loss/1 min. With the modification of original perspiration ratemeter, we constructed a wearable concise ratemeter with a capacitive humidity sensor, a small fan, and a lithium-ion battery in the ventilated capsule.

To inform the self-identified thirst response to the observer, we also constructed a wearable self-identification and self-information apparatus of thirst response with a smartphone.

### Subjects

In total, 16 healthy participants (mean age: 41.6 ± 3.3 years old; eight males and eight females) were enrolled in the present experiments. The body mass index (BMI) of the males and females were 23.7 ± 0.7 and 21.5 ± 0.5, respectively. The minimum number of participants was recommended by the ethical committee for the clinical observation study. Hence, the minimum number we chose was suitable for obtaining a valid conclusion. All the participants provided written and oral informed consent after the detailed explanation and table showing of experimental design, methods, expected results, scientific background and value, compensatory medical tools for harmful damage, and stopping guidelines by the corresponding author. All the data and procedures were confirmed to the tenets of the Declaration of Helsinki. The collected data were stored in Shinshu University School of Medicine with responsibility. All human experiments were conducted in the afternoon from 1:00 to 4:00 pm, considering maximal and stable activity of sympathetic nerve fibers in human circadian rhythm. The temperature and moisture of the examination room were maintained within the range of 22–23 °C and 40–50%, respectively, using air conditioners.

### Experimental protocols

This study was a randomized trial human experiments. A total of 16 healthy participants were enrolled in this study. The subjects were inhibited from water intake and excretion of urine for 1 h before and during the experiments. Immediately before a 30-min step up and down exercise, blood and urine samples were collected from the participants. A wearable perspiration ratemeter and a self-identification and self-information apparatus with a smartphone for thirst response were positioned on their necks and forearms. The step up and down physical exercise lasted for 30 min. The strength of the exercise was approximately 70 Nm, that is, a moderate level, and the average of their pulse rates was approximately 120.9 beats per min. After the 30-min exercise, their blood and urine samples were collected. In addition, their body weights were measured before and after the physical exercise. To evaluate the exercise-induced hemoconcentration, the concentrations of total protein (TP), albumin (Alb), and red blood cells (RBL) in their blood samples were measured by a clinical examination laboratory in Shinshu University Hospital. In addition, to investigate the relationship between the thirst response and changes in the concentration of vasopressin, the concentrations of vasopressin in blood were measured before and after the 30-min physical exercise by SRL Co. Inc. (ISO 15189-accredited by Japan Accreditation Board, RML 00080, Tokyo, Japan). To investigate the relationship between water loss per body surface area and the decreased level of body weight, we used the formula 71.84 × height^0.725^ × weight^0.425^ × 10^−4^^16^. The body mass index (BMI) was also calculated by body weight/body height^2^ (kg/m^2^).

### Statistical analysis

All the data were represented as the mean ± standard errors of the mean. Statistical significance was analyzed using the Student’s t-test for unpaired observations (Microsoft Excel, version 16.54). *p* < 0.05 was considered statistically significant. The relationship between the wearable ratemeter output and water loss in the sweating was compared using linear regression. The Pearson correlation coefficient, r was obtained (Microsoft Excel, version 16.54).

## Results

### Construction of wearable perspiration ratemeter and self-information device of thirst response with a smartphone

#### Wearable perspiration ratemeter for recording physical exercise-mediated sweating

**Figure 1:**
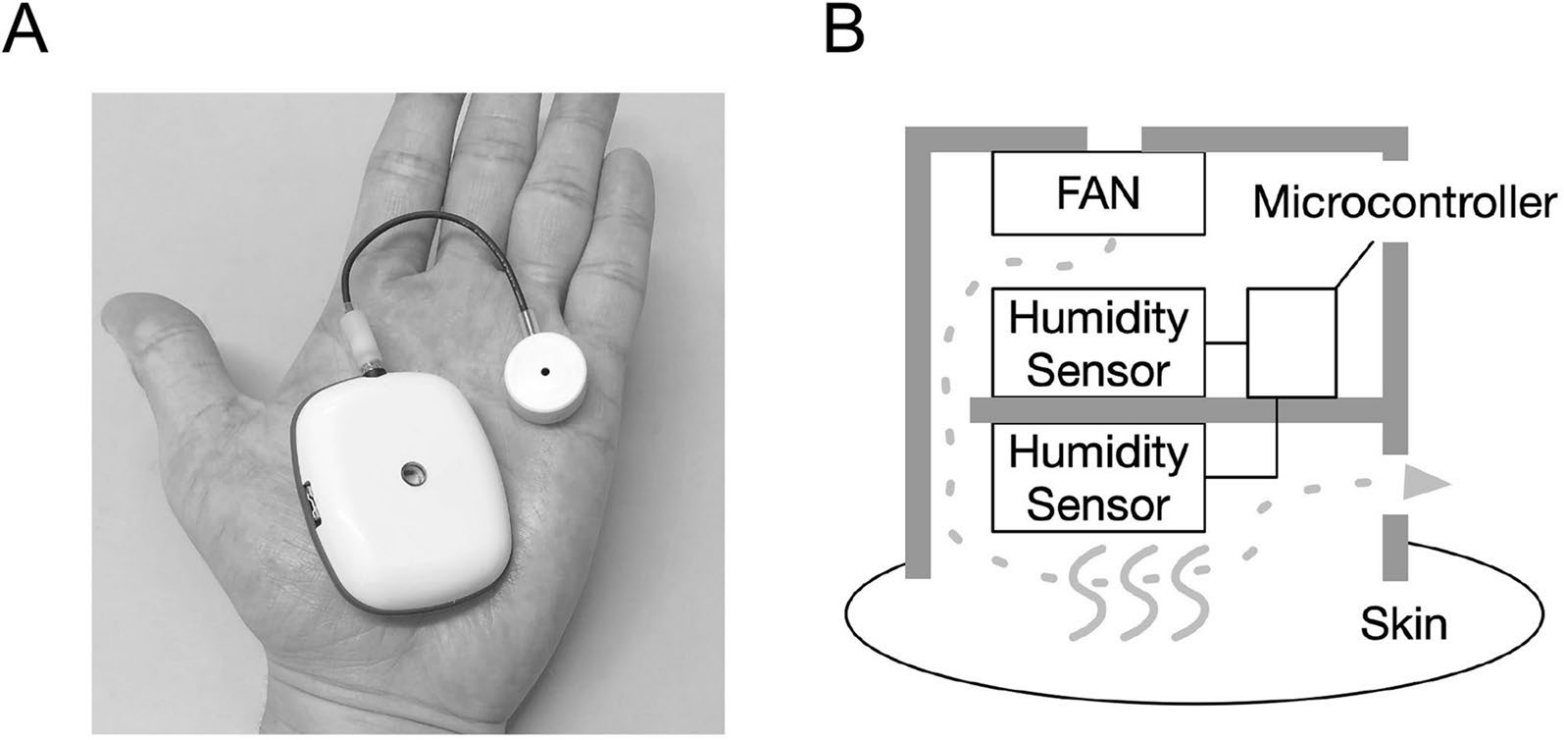
(**A**) Microphotograph of the constructed wearable perspiration ratemeter with ventilated capsule; the box with the lithium-ion battery and microcomputer system in the hand of the participant. (**B**) A schematic of the ventilated capsule with a small fan with the capacitive humidity sensor fixed in each chamber.

To measure large amounts of exercise-induced sweating, we constructed a new wearable perspiration ratemeter (Fig. 1A). It is extremely small (55 mm × 17 mm × 46 mm) and lightweight (35 g). Instead of the airflow circulating system of the original ratemeter, a small fan (UB393-700, Sunon, Japan) is equipped on the top of the ventilated capsule to perfuse air from the upper to the lower chamber, as shown in Fig. 1B. A capacitive humidity sensor (BME280, Bosch, USA) is fixed in each chamber, and a lithium-ion battery is used for the power supply. Both the difference of humidity between the lower and upper chambers and the temperature of the perfused air are calculated in the sweating rate using a microcomputer system. Thus, the absolute amount of water loss per constant time and area of the skin surface is registered on a chart recorder.

**Figure 2:**
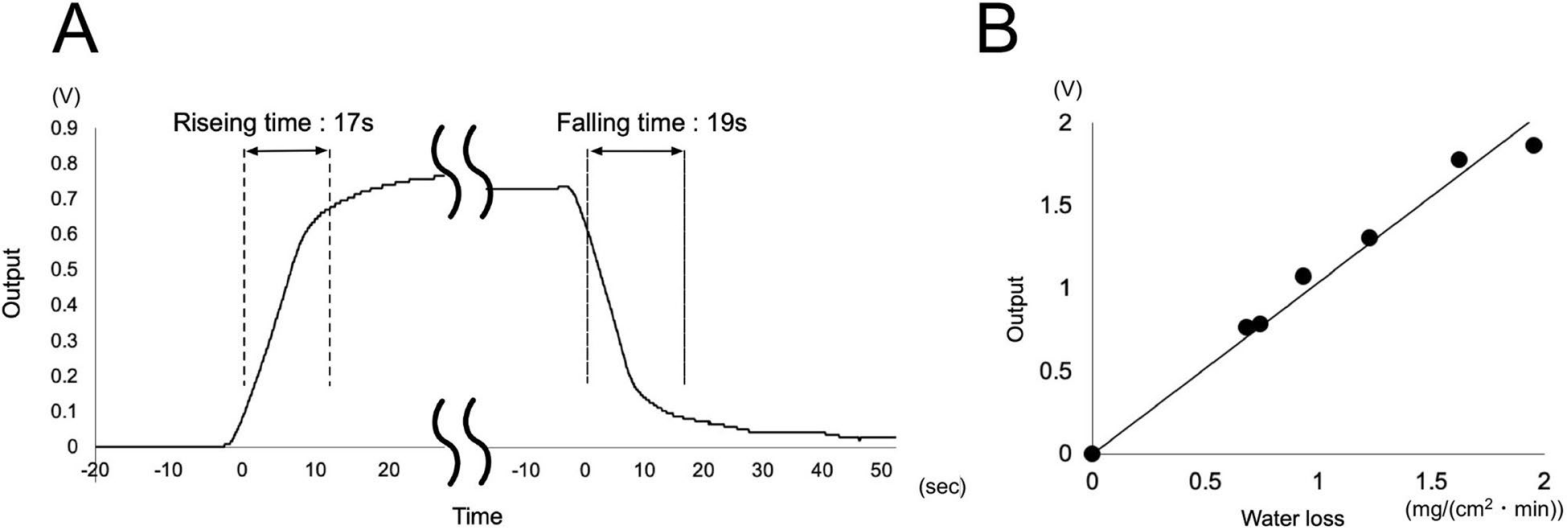
(**A**) Step response curve of wearable sweating ratemeter. Maximum response is obtained at approximately 17 s, and the baseline level regains the initial level after stopping the perfusion of air at 19 s. (**B**) Relationship between electrical output obtained with wearable ratemeter (the ordinate) and amount of water loss over 5 min (abscissa). The sensitivity of the wearable ratemeter is 1.0 V/1 mg/1 min.

Figure 2A illustrates the step responses of the wearable ratemeter in response to starting and stopping the perfusion of air containing a constant volume of water. The method obtained for the step response of wearable ratemeter was used by the same one as the original ratemeter used^14,15^. A maximum response is recorded at approximately 17 s, and the baseline level after stopping the perfusion returns to its initial level at 19 s. Figure 2B shows the relationship between an electrical output obtained with the newly wearable ratemeter and the amount of water loss from a homemade skin model^8^ for 1 min. The correlation factor is 0.995 for water loss ranging from 0 to 2 mg/cm^2^/min. Thus, the sensitivity of the wearable ratemeter is 1.0 V/1 mg/1 min. The method for recording the calibration curve of the wearable ratemeter was the same one as the original ratemeter used^14,15^.

#### Wearable self-identification and self-information apparatus of thirst response

**Figure 3:**
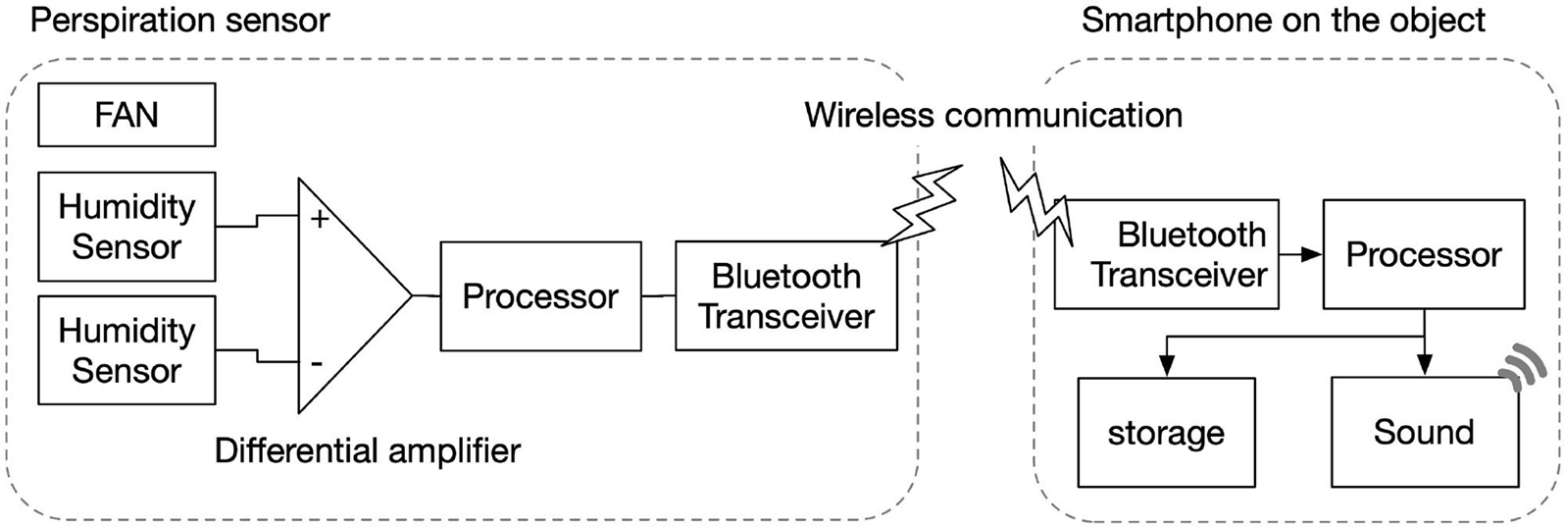
Schematic of informing heatstroke risk to the user with the smartphone. Left panel shows diagram of recording exercise-induced sweating with wearable sweating ratemeter. Right panel shows informing system of heatstroke risk to the user via a sound from the smartphone.

To evaluate the relationship between thirst response and exercise-induced sweating, we constructed a self-identification and self-information apparatus for thirst response during physical exercise using the wearable smartphone. Figure 3 shows the schematic of the apparatus. When the participants were thirsty, they selected a thirst level among three levels of thirst (mild+, middle++, and severe+++) and subsequently touched the level on the display of the smartphone. The apparatus was placed on the participants’ forearms. By using the apparatus, both the level and timing point were recorded simultaneously on the sweating curve of the participants.

#### Device for informing heatstroke risk to the user using a smartphone

By using the timing point to change the slope of sweating curve from an increasing phase to a plateau in the exercise-induced sweating, we constructed a device for informing the heatstroke risk to the users via a sound from the smartphone. The timing point was obtained as follows. The sweating rate average was calculated every 4 min using the curve, and its second derivative was calculated using a personal computer. The timing point was determined as the point whereby the second derivative value was changed from positive to negative. The choice of the timing point as the informing point of the heatstroke risk is shown through human experiments with moderate level exercise.

### Evaluation of the constructed system informing the heatstroke risk in human subjects with physical exercise

To evaluate the validity of timing point for informing the heatstroke risk to users, with human experiments we investigated the relationship between the informing point for the heatstroke risk and the self-identification point of thirst response, and relationship between the self-identification of thirst response and changes in the concentration of vasopressin in blood, urine volume and urine osmolality.

#### Effects of physical exercise on sweating on the neck and thirst level of participants

**Figure 4:**
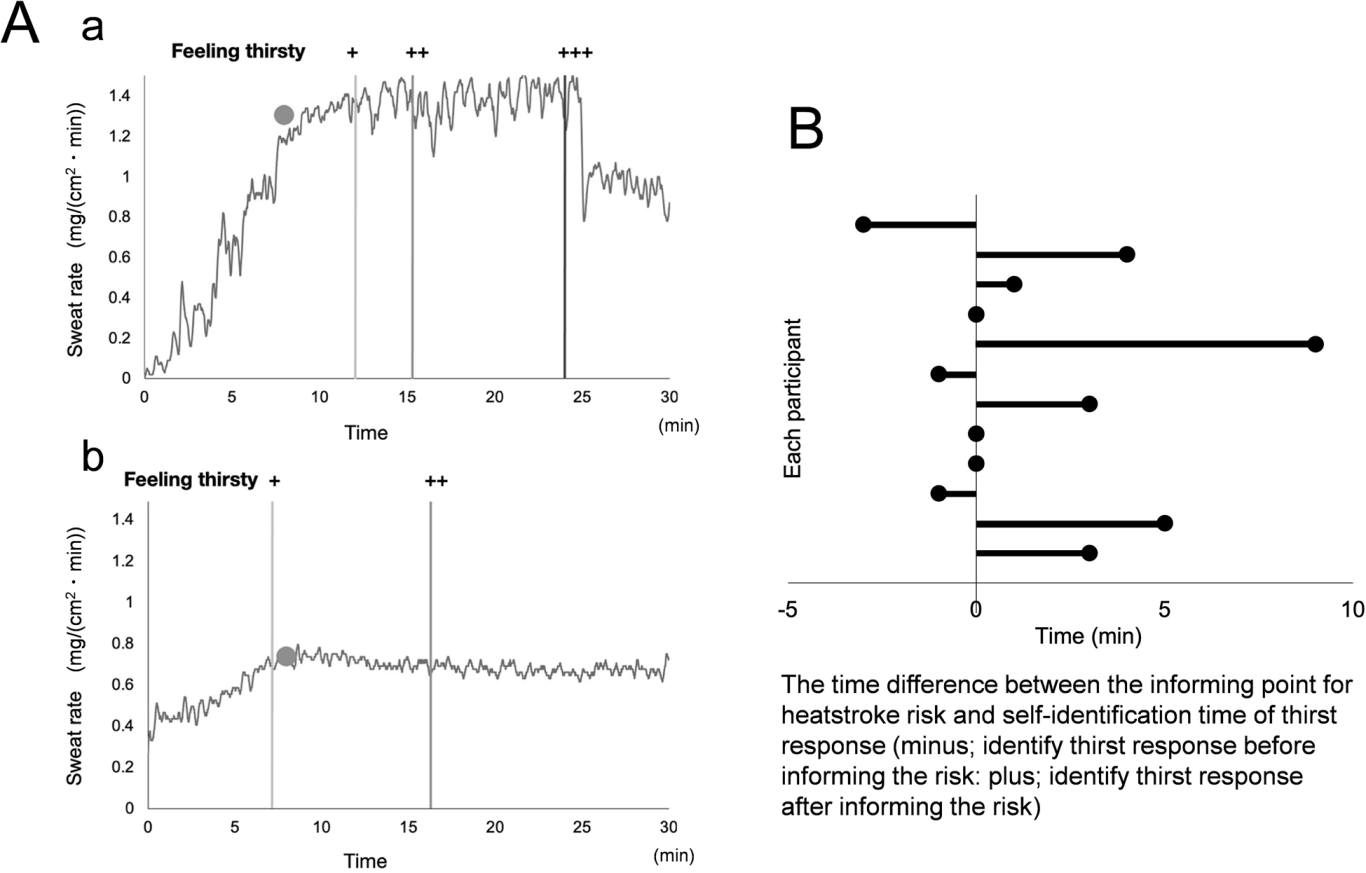
(**A**) Two representative recordings of exercise-induced sweating demonstrating simultaneously the timing points of informing the heatstroke risk (circle) and noticing the feeling thirst (level light+, level middle++, level severe+++). (a) ~ 40 years old female and (b) ~ 40 years old male. (**B**) The relationship between the informing point for heatstroke risk and the self-identification of thirst response in 12 participants. The abscissa shows each participant. The ordinate is the time difference between informing point of heatstroke risk (zero) and self-identification thirst response. The rest 4 participants did not identify the thirst response during the 30 min physical exercise.

Figure 4A illustrates the representative exercise-induced sweating curves measured using the wearable perspiration ratemeter on the necks of two participants: (a) 40-years old female and (b) 40-years old male. In addition, the timing point of the thirst levels is shown using three levels of thirst responses (grade;+, mild;++, strong;+++, severe) on the same sweating curves. The participants were thirsty for several minutes following the informing points of heatstroke risk (●), which were electrically decided as the value of the second derivative of sweating curve changed from positive to negative.

Figure 4B demonstrates the data of 12 participants for the relationship between the informing point for heatstroke risk and the self-identification of thirst response. The rest 4 participants did not identify the thirst response during the 30 min physical exercise. The time point informed the heatstroke risk represents zero in the abscissa of Fig. 4B. The plus and minus values in the abscissa show the time identified thirst response in each participant after and before informing time for heatstroke risk (zero value), respectively. Seven participants identified their thirst response during 0–10 min after the informing time. Only 3 participants noticed the thirst response around 1–3 min before the informing time.

#### Effects of physical exercise on the concentrations of total TP, Alb, RBC, and vasopressin in participants

Table 1 shows the normalized data for the concentrations of total protein (TP), albumin (Alb), red blood cells RBC, and vasopressin in 16 participants obtained before and after the 30 min physical exercise. The concentrations of TP, Alb, and RBC increased slightly with the physical exercise. In contrast, the concentrations of vasopressin in all the participants remarkably increased with the physical exercise.

**Table 1:** The compared data of TP, Alb, RBC, and vasopressin in blood in all participants obtained before and after the 30 min physical exercise.

TP	102.8 ± 0.7% (n = 16), *p* < 0.01
Alb	103.8 ± 0.8% (n = 16), *p* < 0.01
RBC	101.8 ± 0.6% (n = 16), *p* < 0.01
Vasopressin	215.4 ± 29.4%, (n = 16), *p* < 0.01

Each value normalized with one obtained before the exercise.

#### Effects of physical exercise on urine volume and urine osmolarity in participants

**Figure 5:**
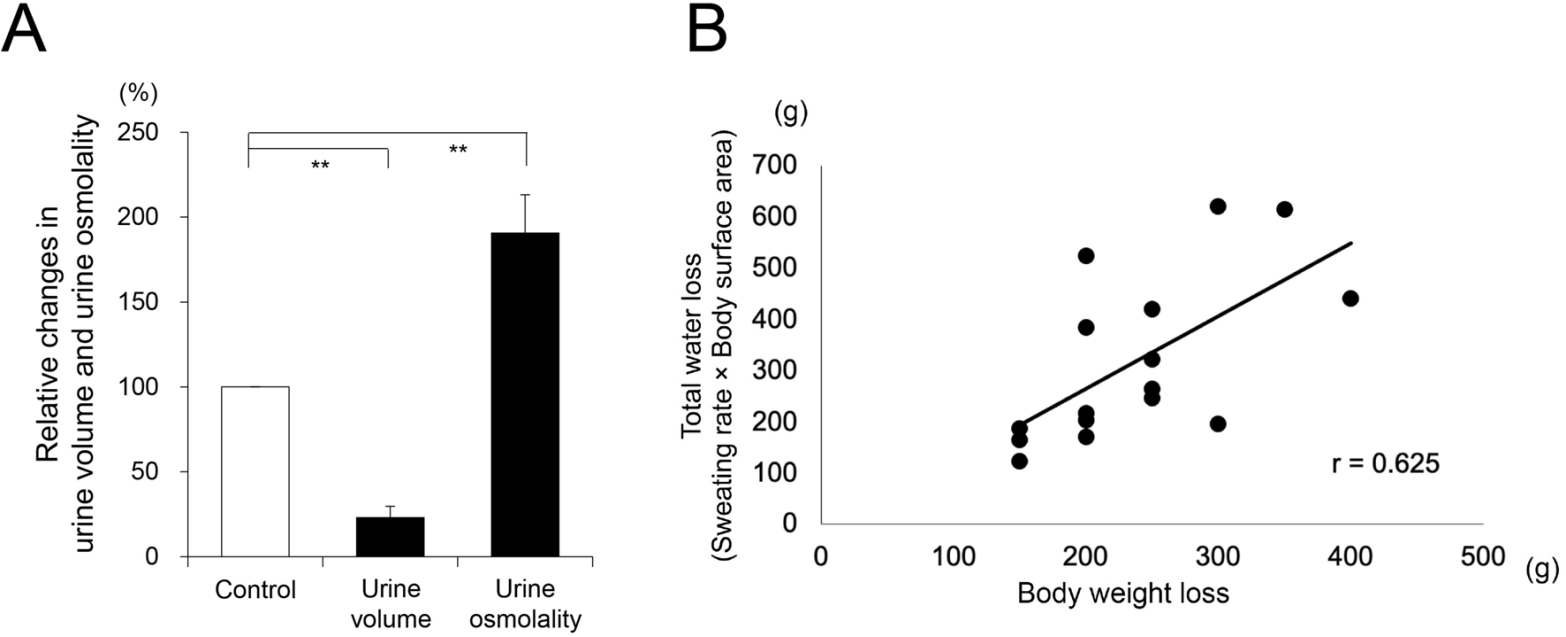
(**A**) Comparison of relative changes in the urine volume and urine osmolality (ordinate) before physical exercise and 30 min after the exercise (n = 16, **p < 0.01). (**B**) Relationship between sweating-mediated water loss and decreased body weight during physical exercise. The total water loss is 269.9 ± 34.3 g (n = 16). The decreased body weight is 262. 5 ± 34.9 g (n = 16).

To clarify whether the increased concentration of vasopressin in the blood is related to the activation of the hypothalamic osmotic center with the increase in the concentration of NaCl in blood, we investigated the effects of physical exercise on urine volume and urine osmolality in 16 participants. Figure 5A demonstrates the normalized data of the urine volume and urine osmolality using the values obtained before and after 30 min physical exercise. The urine volume decreased significantly to 23.0 ± 6.6% of the value obtained before the exercise, *p* < 0.01. In corporation with the findings of urine volume, the urine osmolality increased significantly (190.6 ± 22.5%, *p* < 0.01 vs the value before the exercise).

#### Relationship between changes in body weight, total water loss, and BMI in the participants with physical exercise

Using the body surface area and body mass index (BMI) calculated using the body weight and height of the participants, we investigated the relationships between the sweating-mediated total water loss during the 30 min physical exercise, and changes in body weight and BMI, respectively. The total water loss was 269.9 ± 34.3 g (n = 16). Conversely, the body weight of the participants after the exercise decreased by 262. 5 ± 34.9 g (n = 16). The decreased body weight was approximately similar to the exercise-induced sweating-mediated water loss in the body. Figure 5B shows the relationship between the water loss and the decreased body weight of each participant. In addition, Table 2 demonstrated each value of total water loss, changes in body weight and BMI in all participants. The average of BMI in male and female subjects were 23.73 ± 0.71 and 21.47 ± 0.48 (kg/m^2^), respectively (Table 2).

**Table 2:** The body mass index (BMI, kg/m^2^), total water loss, and body weight loss in all participants with 30 min physical exercise (male n = 8; female n = 8).

No	Male			Female		
	BMI (kg/m^2^)	Sweating rate × Body surface area (g)	Body weight loss (g)	BMI (kg/m^2^)	Sweating rate × Body surface area (g)	Body weight loss (g)
1	20.5	170	150	20.9	164	200
2	26.1	246	250	20.2	186	200
3	23.3	419	400	19.8	523	200
4	25.1	613	300	23.5	196	250
5	21	123	200	22.9	216	150
6	23.9	322	250	20.3	202	250
7	24.9	440	250	21.8	383	400
8	25.1	620	350	22.4	264	300
Mean	23.73	369.12	268.75	21.47	266.75	243.74
SE	0.71	66.48	28.24	0.48	43.92	27.44

The body mass index was calculated by body weight (kg) / body height^2^ (m).

## Discussion

In these experiments, firstly, we constructed a new wearable perspiration ratemeter with the modification of the original one^14,15^. Then, we ensured that the wearable ratemeter is extremely light and concise, thereby suitable for recording exercise-induced or thermal sweating in human subjects. In addition, we verified that the wearable ratemeter have a high sensibility of 1.0 V/1 mg/1 min to detect the exercise-induced sweating although, compared with the original ratemeter for palmar sweating, it had a lower step response. However, no such wearable sweating ratemeter has been available. In the future, we expect to use a wearable perspiration ratemeter to study thermal and exercise-induced sweating and the clinical examination.

On the other hand, various methods have been developed for measurement of human perspiration^17,18^. The total amount of both insensible perspiration and active sweating can be determined by changes in weighing the human body. The method does not, however, offer any possibility of differentiating between evaporative water loss from the skin and respiratory tract. The color-metric methods have been also used, either take colored imprints of sweat drops or to make sweat drops on the skin discernible by staining with colored substances. Among these methods, Minor’s method has been widely used^17^. A disadvantage of the method is that the estimation of degree for active sweating and the judgement of initiation time for active sweating are not always reliable. The simple observation of sweating drops with a magnifying lens was developed for investigating the activity of a single sweat gland^19^. Recently, the ventilated chamber method that a capacitive humidity sensor is detected for moisture of gas flow perfused through the ventilated chamber for human perspiration^20,21^ has been mainly used for perspiration researchers. However, the ventilated chamber method does not become wearable.

On the other hand, the self-identification and self-information wearable apparatus with the smartphone can timely provide the thirst response based on sweat recording in the participants. The self-information wearable apparatus may be useful, for instance, in schools to evaluate the thirst response of students during physical exercises or in old people’s homes to determine the thirst response of those who are bedridden.

Another important aspect of the experiment is the construction of the device to inform the users of heatstroke risk by using the smartphone. Based on the human experiments, we set the timing point for informing heatstroke risk as the point whereby the second derivative value of the 4-min sweating rate was changed from positive to negative. In other words, the timing point is the point whereby the slope of the sweating curve is changed from an increasing phase to a near plateau phase. Additionally, it is the starting point for a decrease in the sweating rate, which may be coincided with the starting point of an exercise-mediated hemoconcentration. Because almost participants were identified several min after informing heatstroke risk with the smartphone, being related to the hemoconcentration-mediated activation of osmoreceptors in the hypothalamus. Thus, the hemoconcentration of TP, Alb, and RBC within the range of 101–103% (Table 1) is approximately the same as that calculated using the sweating-mediated total water loss (~ 270 g) and the weight of circulatory blood volume (~ 5 kg). In contrast, the findings that the increased concentration of vasopressin in the blood (~ 215%) was higher than those of TP, Alb, and RBC may be related to the stimulation of an osmoreceptor in the hypothalamus and the release of vasopressin from the posterior pituitary gland. In agreement with this evidence, thirst responses were observed in most of the participants during the exercise. Notably, all the participants produced a significant decrease in urine volume with an increase in urine osmolality. Thus, the thirst response may be produced by the activation of an osmoreceptor in the hypothalamus. The increase of thirst response physiologically ordered human subjects to the need for drinking water, thus preventing hemoconcentration in corporation with a vasopressin-mediated increase in urine absorption on a negative feedback system.

Several studies on the advantages of fluid ingestion on thermoregulatory and cardiovascular responses during progressive dehydration-related hemoconcentration have been reported^22,23^. However, the contribution of exercise-induced sweating in dehydration-related hemoconcentration and information systems for heatstroke risks, to the best of our knowledge, has not yet been evaluated. Therefore, we developed a wearable sweating ratemeter for informing the users of heatstroke risk. The constructed system will be needed in the future to evaluate in detail with additional clinical experiments. Especially, we should reevaluate, in the future, the suitability to decide the informing point for heatstroke risk.

## Data Availability

All relevant data are available from the corresponding author on request.

## References

[1] Epstein Y, Yanovich R (2019). Heatstroke. N. Engl. J. Med..

[2] Bouchama A, Knochel JB (2002). Heat stroke. N. Engl. J. Med..

[3] Falk B, Dontan R (2008). Children’s thermoregulation during exercise in the heat: A revisit. Appl. Physiol. Nutr. Metab..

[4] Epstein Y (1999). Heat stroke: A case series. Med. Sci. Sports Exerc..

[5] Shibasaki M, Crandall CG (2010). Mechanisms and contollers of eccrine sweating in humans. Front. Biosci..

[6] Cheshire W, Freeman R (2003). Disorders of sweating. Semin. Neurol..

[7] Biglia N (2017). Vasomotor symptoms in menopause: A biomarker of cardiovascular disease risk and other chronic diseases?. Climacteric.

[8] Homma S (2001). Hippocampus in relation to mental sweating responses evoked by memory recall and mental calculation: A human electroencephalography study with dipole tracing. Neurosci. Lett..

[9] Homma S (1998). Intracerebral source localization of mental process-related potentials elicited prior to mental sweating responses in humans. Neurosci. Lett..

[10] Asahina M (2003). Emotional sweating response in a patient with bilateral amygdala damage. Int. J. Psychophysiol..

[11] Momose H (2020). Eyes closing and drowsiness in human subjects decrease baseline galvanic skin response and active palmar sweating: Relationship between galvanic skin and palmar perspiration responses. Front. Physiol..

[12] Seekl, J.R. *et al.* Oral hypertonic saline causes transient fall of vasopressin in humans. *Am. J. Physiol.* **251**(Regulatory Integrative Comp. Physiol. 20), R214–R217 (1986). 10.1152/ajpregu.1986.251.2.R214 3740301

[13] Thompson, C. J. *et al.* Acute suppression of plasma vasopressin and thirst after drinking hypernatremic humans. *Am. J. Physiol.* **252**(Regulatory Integrative Comp. Physiol. 21), R1138–R1142 (1987). 10.1152/ajpregu.1987.252.6.R1138 3591984

[14] Sakaguchi M (1988). A new apparatus for continuous recording of sweating rate by using a hygrometer. Jpn. J. Med. Eng..

[15] Ohhashi (1998). Human perspiration measurement. Physiol. Meas..

[16] Duboism D, Dubois EF (1916). A formula to estimate the approximate surface area if height and weight be known. Arch. Intern. Med..

[17] Kuno Y (1956). human Perspiration.

[18] Nilsson GE, Öberg PA, Rolfe P (1979). Measurement of evaporative water loss: Methods and clinical applications. Non-invasive Physiological Measurement.

[19] Kamei T, Naitoh K, Nakashima K, Ohhashi T, Kitagawa S, Tuda T (1997). Instrumentation of a handy microscopic probe for concurrent observation and measurement of active sweat secretion, and its application. J. Pharmacol. Biomed. Anal..

[20] Bullard RW (1962). Continuous recording of sweating rate. J. Appl. Physiol..

[21] Ogawa T (1970). Local effect of skin temperature on threshold concentration of sudorific agents. J. Appl. Physiol..

[22] Takamata, A. *et al.* Osmoregulatory modulation of thermal sweating in humans: Reflex effects of drinking. *Am. J. Physiol.* **268**(Regulatory Integrative Comp. Physiol. 37), R414–R422 (1995). 10.1152/ajpregu.1995.268.2.R414 7864236

[23] Coyle EF, Montain E, Gisolfi CV, Lamb DR, Nadel ER (1993). Thermal and cardiovascular responses to fluid replacement during exercise. Perspectives in Exercise Science and Sports Medicine. Exercise, Heat, and Thermoregulation.

